# Cholesterol Concentration in Cell Membranes and its Impact on Receptor–Ligand Interaction: A Computational Study of ATP-Sensitive Potassium Channels and ATP Binding

**DOI:** 10.1007/s00232-025-00345-4

**Published:** 2025-03-26

**Authors:** Cesar Millan-Pacheco, Iris N. Serratos, Gerardo J. Félix-Martínez, Gerardo Blancas-Flores, Alejandra Osorno, Rafael Godínez

**Affiliations:** 1 Facultad de Farmacia, Universidad Autónoma del Estado de Morelos, Morelos. Av. Universidad No. 1001, Colonia Chamilpa, 62209 Morelos, México; 2 Departamento de Química, Universidad Autónoma Metropolitana-Iztapalapa, San Rafael Atlixco 186, Col. Vicentina. Iztapalapa, C. P. 09340 Ciudad de Mexico, México; 3 Departamento de Ingeniería Eléctrica, Universidad Autónoma Metropolitana-Iztapalapa, San Rafael Atlixco 186, Col. Vicentina. Iztapalapa, C. P. 09340 Ciudad de Mexico, México; 4 Departamento de Ciencias de la Salud, Universidad Autónoma Metropolitana, Unidad Iztapalapa, Av. San Rafael Atlixco 186, Col. Vicentina, Iztapalapa, C. P. 09340 Ciudad de Mexico, México

**Keywords:** Adenosine triphosphate (ATP), ATP-dependent potassium channels (K_ATP_), Molecular recognition, Pancreatic beta-cells, Cholesterol percentage, Computational approaches

## Abstract

This work describes a computer study that looks at how different amounts of cholesterol (0%, 25%, and 50%) in cell membranes change the relationship between ATP and the K_ATP_ channel. This could explain why pancreatic beta-cells secrete insulin differently. We use computer simulations of molecular dynamics, calculations of binding free energy, and an integrated oscillator model to look at the electrical activity of beta-cells. There is a need for this kind of multiscale approach right now because cholesterol plays a part in metabolic syndrome and early type 2 diabetes. Our results showed that the increase in cholesterol concentration in the cell membrane affects the electrostatic interactions between ATP and the K_ATP_ channel, especially with charged residues in the binding site. Cholesterol can influence the properties of a membrane, including its local charge distribution near the channel. This affects the electrostatic environment around the ATP-binding site, increasing  the affinity of ATP for the channel as our results indicated from 0 to 25 and 50% cholesterol (− 141 to − 113 kJ/mol, respectively). Simulating this change in the affinity to ATP of the K_ATP_ channels in a model of the electrical activity of the pancreatic beta-cell indicates that even a minimal increase could produce hyperinsulism. The study answers an important research question about how the structure of the membrane affects the function of K_ATP_ and, in turn, insulin releases a common feature of metabolic syndrome and early stages of type 2 diabetes.

**Graphical Abstract:**

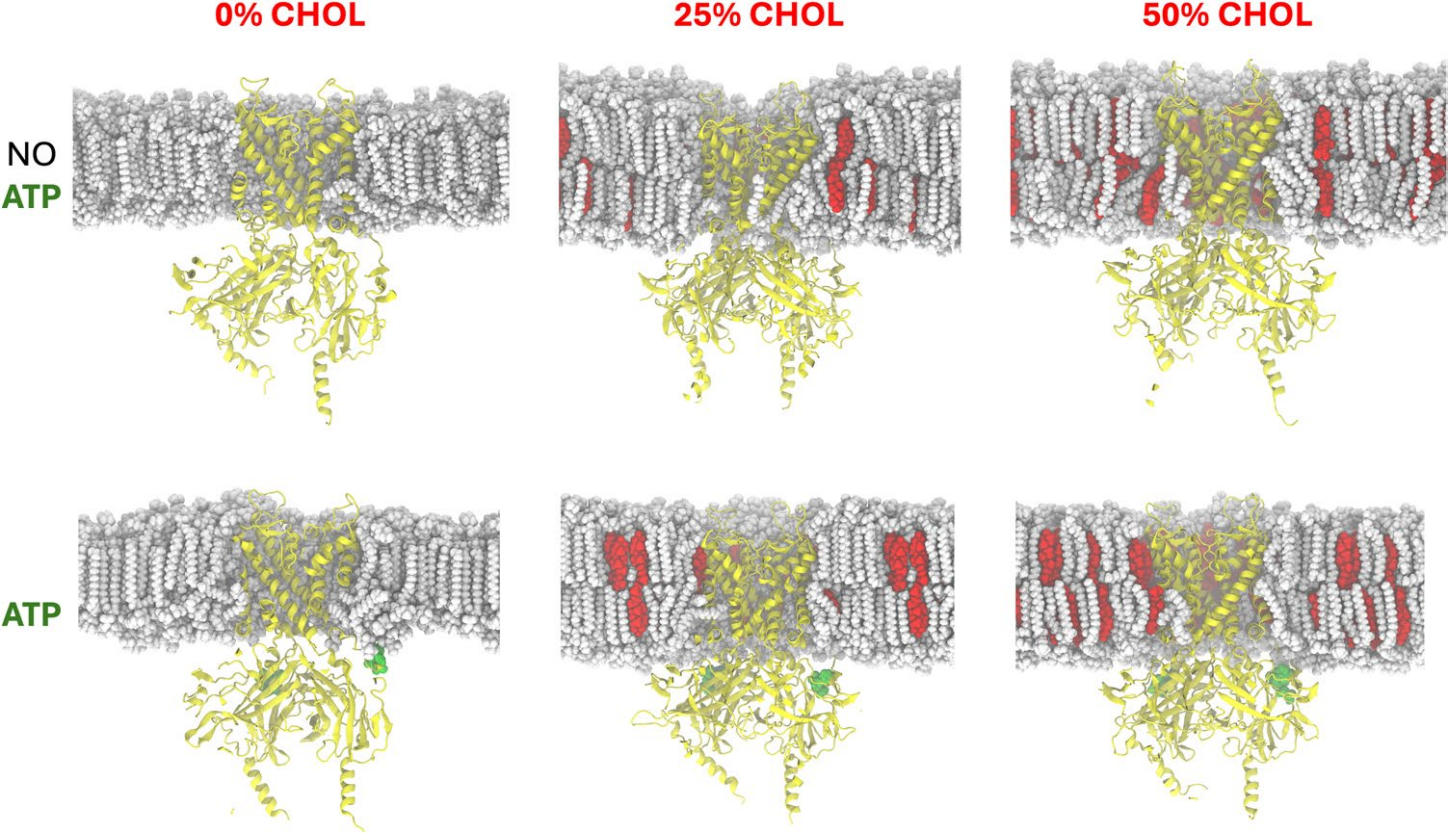

## Introduction

In pancreatic beta-cells, the adenosine triphosphate (ATP)-sensitive potassium channels (K_ATP_) play a crucial role in linking cellular metabolism to electrical excitability and insulin secretion. The opening and closing of K_ATP_ in response to changes in the cellular ATP/ADP ratio regulates the membrane potential, which reflects the metabolic state of the cell (Rorsman and Ashcroft 2018). Under normal healthy conditions, when cellular ATP levels rise, as occurs during glucose stimulation, K_ATP_ channels close, leading to membrane depolarization, triggering the electrical activity of the cell. This, in turn, leads to the opening of voltage-gated calcium channels and an increase in intracellular calcium concentration. This calcium influx subsequently triggers exocytosis of insulin granules. Conversely, when ATP levels decrease, K_ATP_ open, causing membrane polarization and reduced calcium influx, thereby inhibiting insulin secretion (Ashcroft 2023). Therefore, the closure of K_ATP_ in response to an increase in ATP is a critical factor in determining the electrical activity and calcium signaling dynamics underlying the secretion of insulin. Accordingly, alterations in K_ATP_ have significant consequences on pancreatic beta-cell function (Remedi and Nichols 2016).

Furthermore, obesity, dyslipidemias, hypertension, elevated fasting blood glucose, insulin resistance, and hyperinsulinemia, collectively known as metabolic syndrome, are major risk factors for the development of type 2 diabetes (Fahed et al. 2022). Although hyperinsulinemia could result from the overstimulation of insulin secretion from pancreatic beta-cells due to insulin resistance, van Vliet et al. (2020) reported that insulin secretion, both during basal conditions and after glucose ingestion, is increased in individuals with obesity even in the absence of insulin resistance. However, the mechanism underlying this abnormality remains debatable. Recent studies have emphasized the important role of cholesterol in beta-cell function. Notably, cholesterol significantly influences the physical properties of the cell membrane, and consequently, the distribution and function of membrane proteins, including ionic channels (Galli et al. 2023; Perego et al. 2019; Levitan et al. 2014). Here, we propose that the altered sensitivity of K_ATP_ to ATP, resulting from changes in the cholesterol composition of the pancreatic beta-cell membrane, may contribute to the development of the hyperinsulinemic environment observed in the early stages of metabolic dysfunction, as is the case in obesity and metabolic syndrome. Hao et al. (2007) and Pihlajamäki et al. (2004) suggested that over time, increased cholesterol affects the functioning of pancreatic beta-cells. Initially, the cell responds appropriately by increasing insulin secretion (hyperinsulinism). However, prolonged cholesterol exposure leads to metabolic dysfunction, inflammation, and cellular damage, ultimately reducing the beta-cells’ ability to secrete insulin. Xiao et al. (2022) mentioned that a chronic increase in cholesterol and glucose can cause insulin resistance and dysfunction of pancreatic beta-cells caused by the alteration of insulin and glucose signaling pathways, which affect cholesterol synthesis and secretion of insulin. This long-term detrimental effect on beta-cell function explains why elevated cholesterol results in decreased insulin secretion rather than hyperinsulinism in more advanced stages of the disease.

Cholesterol is an organic molecule that plays a crucial role as a structural component of the mammalian plasma membrane (Brown and London 2000). Cholesterol and phospholipid levels modulate the fluidity of cell membranes. Alterations in cholesterol concentration can contribute to the development of various diseases, including diabetes (Cho et al. 2009). In recent years, several research groups have carried out experimental studies and molecular dynamics simulations regarding the behavior of phospholipids and cholesterol in membranes (Ohvo-Rekilä et al. 2002; Robinson et al. 1995; Gabdouline et al. 1996; Liu et al. 1998; Baker and Abrams 2014). Numerous studies have listed the effects of cholesterol on various types of ion channels, including potassium channels, which are sensitive to increases in membrane cholesterol (Levitan et al. 2014; Levitan et al. 2010; Romanenko et al. 2004; Romanenko et al. 2002; Romanenko et al. 2014; Rosenhouse-Dantsker et al. 2011; Jiang and Levitan 2022).

In this study, we focused particularly on K_ATP_, which are primarily composed of Kir6.2 and the sulfonylurea receptor-1 (SUR1), both of which are highly expressed in pancreatic beta-cells (Principalli et al. 2015). Fortunately, K_ATP_ (Li et al. 2017; Wu et al. 2018) have been deposited in the Protein Data Bank (PDB). Recently, Driggers et al. (2024) reported the cryo-EM structure of a K_ATP_ harboring the neonatal diabetes mutation Kir6.2-Q52R in the open conformation-bound PI(4,5)P2 (PIP2), which disfavors ATP binding. Similar changes have been described in the pre-open structure of the SUR1-Kir6.2^H175K^ fusion channel (Wang et al. 2022). We used the cryo-EM structure of the pancreatic K_ATP_ bound to ATP at 3.41 Å resolution reported previously (Sung et al. 2022). In addition, this is one of the most complete structures, with better resolution than other crystals, and mainly reveals the details of the ATP-binding sites.

On the other hand, blood glucose levels increase when humans consume food. Through a transporter, glucose enters pancreatic beta-cells, where it is primarily converted to ATP via glycolysis. The increase in ATP within beta-cells promotes its binding to the K_ATP_, consequently leading to channel closure. Under physiological conditions, when blood glucose levels decrease, beta-cell do not generate electrical activity and maintain a resting potential of approximately ~ -65 mV. If the ATP-sensitive potassium channel remains closed, the resting potential becomes less negative, a process known as depolarization. In response to depolarization, calcium channels open, allowing calcium to enter in the cell. This influx of calcium triggers exocytosis, leading to the secretion of insulin, which is subsequently released into the bloodstream. Secreted insulin lowers blood sugar levels, reducing the amount of glucose entering the beta-cells. Therefore, ATP production is low, closing  the K_ATP_, that is, the channel opens again, and the beta-cell returns to its resting potential of ~ -65 mV (repolarization), ceasing the electrical activity of the cell and favoring the release of insulin.

In this study, we analyzed the cryo-EM structure of the K_ATP_ (ID PDB 7TYS) (Sung et al. 2022), however, we used Kir6.2 with and without ATP for in silico studies. We tested the conservation of the binding site and subsequently conducted clustering analysis using molecular dynamics. All structures were embedded in a cell membrane with varying concentrations of cholesterol: 0% as control, 25% representing physiological conditions, and 50% reflecting alterations, such as obesity. These models enabled us to assess both the electrostatic and non-electrostatic contributions to the free binding energy. To complement these studies, we conducted simulations of the electrical activity of pancreatic beta-cells with an increased affinity to ATP of the K_ATP_, aiming to contribute to elucidating the molecular mechanisms underlying hyperinsulinism, an alteration commonly observed in metabolic syndrome and early stages of type 2 diabetes. Furthermore, the study was carried out with the objective of understanding whether variations in the composition of lipids in the membrane (specifically cholesterol) directly affect the electrostatic interactions between ATP and the K_ATP_ channel by changing the membrane’s physical properties, which in turn alters the electrostatic environment around the channel and the ATP-binding process.

## Materials and Methods

### Structure Preparation: K_ATP_ and ATP

#### Molecular Dynamics Simulations

The *Rattus norvegicus* pancreatic Kir6.2 (PDB ID: 7TYS) (Sung et al. 2022) was simulated with and without ATP molecules bound. The channel was embedded in a 1,2-dipalmitoylphosphatidylcholine (DPPC) membrane, and simulations were carried out at three distinct cholesterol concentrations (0, 25, and 50%). The cholesterol concentration is known by its capacity to modify the membrane dynamics. Cholesterol concentration was chosen to study the behavior of the Kir6.2 on these membrane conditions. As reported by Mardešić, cholesterol concentrations have ranges from 10 to 30 mol% and in some cases it can be as high as 66 mol% (Mardešić 2023). The orientation of the protein channel was obtained from the Orientation of Proteins in Membranes database (Lomize et al. 2012, 2022), utilizing the corresponding input generator option available on the CHARMM-GUI (Jo et al. 2008; Wu et al. 2014) server. All systems were prepared with 0.15M KCl and sufficient water to ensure complete solvation. The input parameters recommended by the CHARMM-GUI server were as follows: All systems were simulated for 100 ns in triplicate using GROMACS 2019 (Berendsen et al. 1995; Páll et al. 2020) with CHARMM36 force field parameters (Huang and MacKerell 2013; Huang et al. 2017). Charmm36 force field was used due to its capability to model lipid bilayers. There are multiple articles that showed that charmm36 potential is able to handle membrane systems that include protein/membrane, sterols/membrane and realistic membranes (Jo et al. 2009; Klauda et al. 2012; Lim et al. 2012; Sandoval-Perez et al. 2017; Marrink et al. 2019). Molecular dynamics analyses were performed using GROMACS built-in tools and custom Linux scripts. Visualization and image generation were performed using Visual Molecular Dynamics (VMD) (Humphrey et al. 1996).

#### Binding Free Energy Calculations (ΔG_b_)

Cluster analyses were performed during the final 40 ns of each simulation. These complexes allow us to determine the electrostatic and non-electrostatic contributions to the ΔG_b_ between ATP and Kir6.2 obtained at different amounts of cholesterol (0%, 25%, and 50%) in cell membranes. Cholesterol can affect the local electrostatic environment of the membrane, including the distribution of charged lipids. These lipids could alter the charge density and the spatial distribution of charges around the Kir6.2 channel, influencing how ATP binds.

The ΔG_b_ values were estimated using the method described by Baker et al*.* (Baker et al. 2001). Binding energies were calculated using the following equations:

1 $$\Delta {\text{G}}_{{\text{b}}} = \Delta {\text{G}}_{{{\text{solv}}}} + \Delta {\text{G}}_{{{\text{Coul}}}} + \Delta G_{{{\text{non}} - {\text{elec}}}},$$

where ΔG_b_ is the binding energy, ΔG_solv_ represents the solvation energy, and ΔG_Coul_ corresponds to the Coulombic energy, both of which comprise the electrostatic component. ΔG_non-elec_ accounts for the non-polar contribution to the overall binding energy.

Electrostatic contributions were calculated using an implicit solvent model. Dielectric constants of 78 and 4 were applied to water and Kir6.2, respectively, using the Adaptive Poisson–Boltzmann solver (APBS) program (http://www.poissonboltzmann.org/) (Jurrus et al. 2018). For Kir6.2 channel, ionic radii and atomic charges were assigned based on the CHARMM force field (MacKerell et al. 1998). The protonation states of ionizable residues at pH 7.0 were determined using PROPKA (Søndergaard et al. 2011; Dolinsky et al. 2004). Both the CHARMM force field and PROPKA were integrated into the PDB2PQR server (http://server.poissonboltzmann.org/pdb2pqr) (Dolinsky et al. 2004). The ATP atomic charges were assigned using the force field implemented in the AutoDock Vina program (Trott and Olson 2010). Non-electrostatic contributions were calculated by multiplying the change in solvent-accessible surface area (ΔASA) upon binding by the coefficient γ, an interfacial tension value of 0.021 kJ/mol*Ǻ^2^ ( Levy et al. 2003), using the following equation:

2 $$\Delta G_{non-elec} = \gamma \left( {ASA_{Kir6.2 - ATP} - ASA_{Kir6.2} - ASA_{ATP} } \right)$$

ΔASA changes were obtained using the VMD software (Humphrey et al. 1996).

It is important to mention that these ΔG_b_ calculations were determined by modifying the cholesterol in the cell membrane, where important information was obtained on the displacements and shifts due to the increase in cholesterol. However, the challenge remains to determine ΔG_b_ values by molecular dynamics that are dependent on ATP concentrations for a better refinement of the values. Likewise, the APBS algorithm is an implicit model that simulates the electric field generated by charges in a continuous medium. In this type of model, the solvent or surrounding medium is uniformly represented by an effective dielectric constant instead of explicitly modeling the solvent molecules as in molecular dynamics simulations. An implicit dielectric constant in the model simplifies calculations by not having to simulate the solvent’s atomic-level interactions, but it maintains the essence of polarization and screening of electrostatic interactions.

#### Simulation of the Effect of Increased K_ATP_ Affinity to ATP on Insulin Secretion of the Pancreatic Βeta-Cell

The function of pancreatic beta-cells was simulated using the Integrated Oscillation Model (IOM) (Bertram et al. 2023; McKenna et al. 2016), which incorporates a metabolic component coupled to components for calcium handling and electrical activity. The IOM proposes that glucose metabolism drives the production of ATP, leading to the closure of K_ATP_, which results in the depolarization necessary for generating electrical activity through the interaction of potassium, sodium, and calcium channels. This ultimately increases the intracellular calcium concentration (Fig. 6a). In the IOM model, ATP is consumed through both hydrolysis and the activity of calcium pumps located in the cell membrane and endoplasmic reticulum, whereas metabolism is further stimulated by an increase in intracellular calcium. Because elevated intracellular calcium is widely recognized to promote insulin secretion, we used intracellular calcium levels as an indicator of insulin secretion. The IOM model was chosen to evaluate the effect of increased K_ATP_ affinity on beta-cell function, as it can replicate key experimental findings, including a wide range of electrical bursting patterns, calcium oscillations, metabolic fluctuations, and oscillations in K_ATP_ conductance. To simulate the increased K_ATP_ affinity, we decreased the dissociation constant (Ktt) associated with ATP binding to the channel, following the percentage changes estimated from the molecular simulations described above. That is, based on the ΔG_b_ values reported in Table 1, it allowed us to determine the affinity constants through K = exp(-ΔG_b_ /(RT)) for each simulation and all the cases considered (0, 25, and 50% cholesterol) with R = 8.314 kJ/(mol K) and T = 298.15 K. Average affinity constant decreased from 0.979 ± 0.028 (0% cholesterol) to 0.942 ± 0.055 (25% cholesterol) and 0.968 ± 0.020 (50% cholesterol), representing an approximate decrease of 3.85% and 1.15%, respectively. Based on these estimations, a 4% increase in the affinity of the K_ATP_ was adopted in the model of the electrical activity of the pancreatic beta-cell by modifying the value of the Ktt dissociation constant from 1 mM to 0.96 mM in the IOM model. Beyond decreasing the ATP dissociation constant, the IOM model was implemented without further modification as previously described (Marinelli et al. 2022). Simulations of beta-cell electrical activity were conducted using XPPAUT (Ermentrout and Mahajan 2003).

**Table 1:** ΔG_b_ values of the cryo-EM structure of Kir6.2 (7TYS) (Sung et al. 2022), with ATP determined at pH 7.0 by APBS (Jurrus et al. 2018) and VMD1.9.1 (Humphrey et al. 1996). Complexes were obtained by previous docking studies based on the representative structure from each cluster of the molecular dynamics trajectories

Cholesterol percentage	Kir6.2 channel-ATP	ΔG_solv_ (kJ/mol)	ΔG_Coul_ (kJ/mol)	ΔG_non-elec_ (kJ/mol)	ΔG_b_^*^ (kJ/mol)
0%	Cluster1	231	73	− 52	106
	Cluster2	251	− 112	− 58	81
	Cluster3	264	− 238	− 55	− 29
	**Mean**	**249(17)**	**− 141(86)**	**− 55(3)**	**53(72)**
25%	Cluster1	299	− 171	− 60	68
	Cluster2	326	− 54	− 56	216
	Cluster3	239	− 115	− 60	64
	**Mean**	**288(45)**	**− 113(59)**	**− 59(2)**	**116(87)**
50%	Cluster1	243	− 162	− 56	25
	Cluster2	240	− 54	− 59	127
	Cluster3	271	− 122	− 59	90
	**Mean**	**251(17)**	**− 113(55)**	**− 58(2)**	**81(52)**

^*^The ΔG_b_ is given by Eq. (1). Numbers in parentheses are standard deviations

## Results and Discussion

### Molecular Dynamics Simulations

**Fig. 1:**
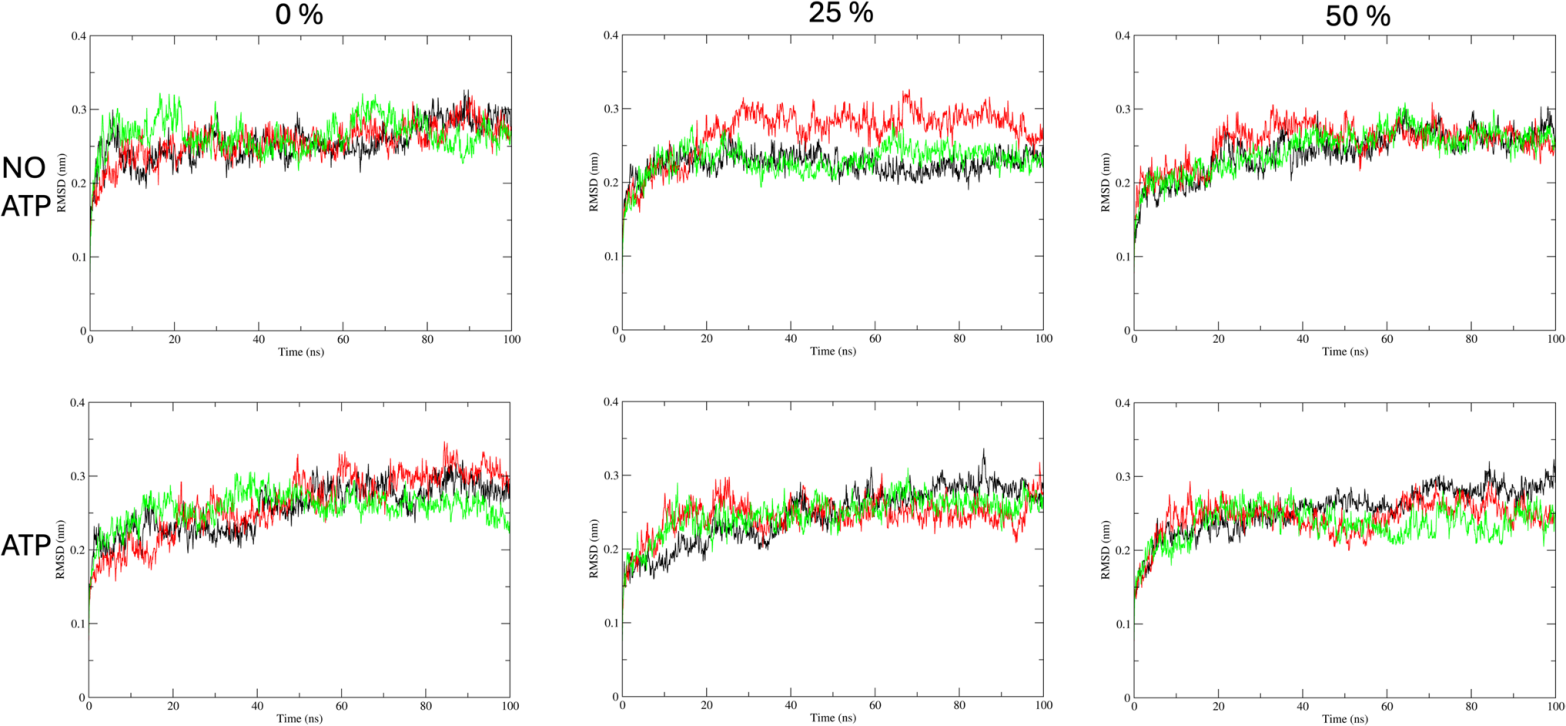
RMSD of the α-carbon of the Kir6.2 over time during simulations with and without ATP

Molecular dynamics simulations of the Kir6.2 (with and without ATP) were conducted at three different cholesterol concentrations to evaluate the dynamics of the pores over time. All molecular simulations were performed on DPPC membranes with three distinct cholesterol concentrations (0, 25, and 50%), each in triplicate. The root means square deviations (RMSD) of the α-carbons indicated that the systems stabilized over time, although at different stages (Fig. 1). In the simulations without cholesterol, the systems stabilized after approximately 50 ns, whereas the 25 and 50% cholesterol simulations reached stability after approximately 20 ns. This difference may be attributed to the well-known role of cholesterol as a membrane stabilizer, suggesting that stabilization of the membrane environment around the channel could influence its behavior.

**Fig. 2:**
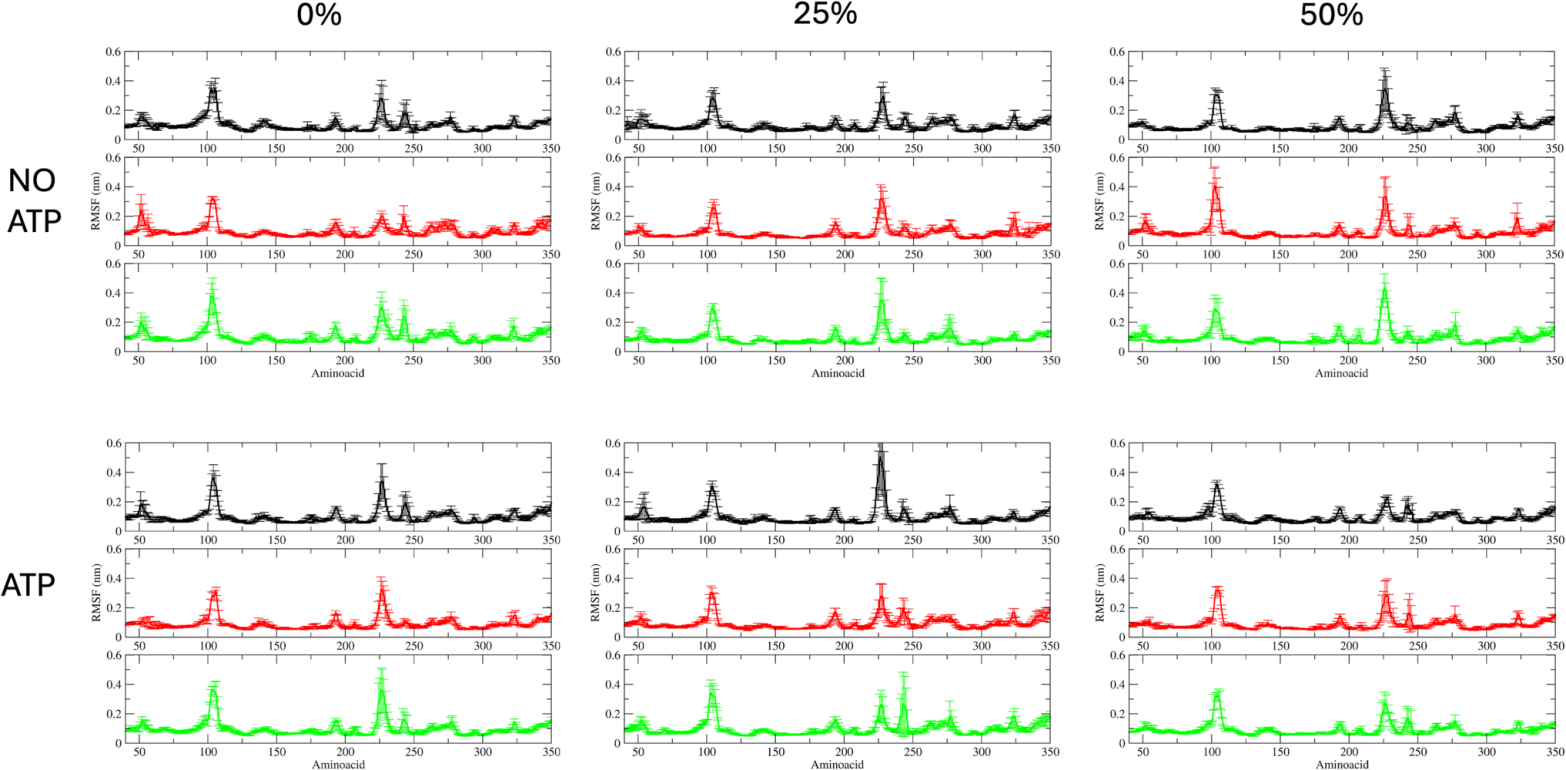
RMSF by residue for each system studied in this study. Kir6.2 is a tetramer so for each replica the average and standard deviation are shown (Replica 1 on black, replica 2 on red, and replica 3 on green). RMSF values for the first 10 (amino terminal) and the last 11 amino acids (carboxyl terminal) were not shown due that the values for these residues are expected to have more movement than the rest of the protein

One way to analyze the cholesterol concentration impact on the protein dynamics is to measure the fluctuations of the protein against a reference structure. The root mean square fluctuation (RMSF) is a measurement of how the atomic positions move from a reference position over time. We used as reference the center of the most populated cluster of similar structures over the last 40 ns for each system. The RMSF for each system is shown in Fig. 2.

**Fig. 3:**
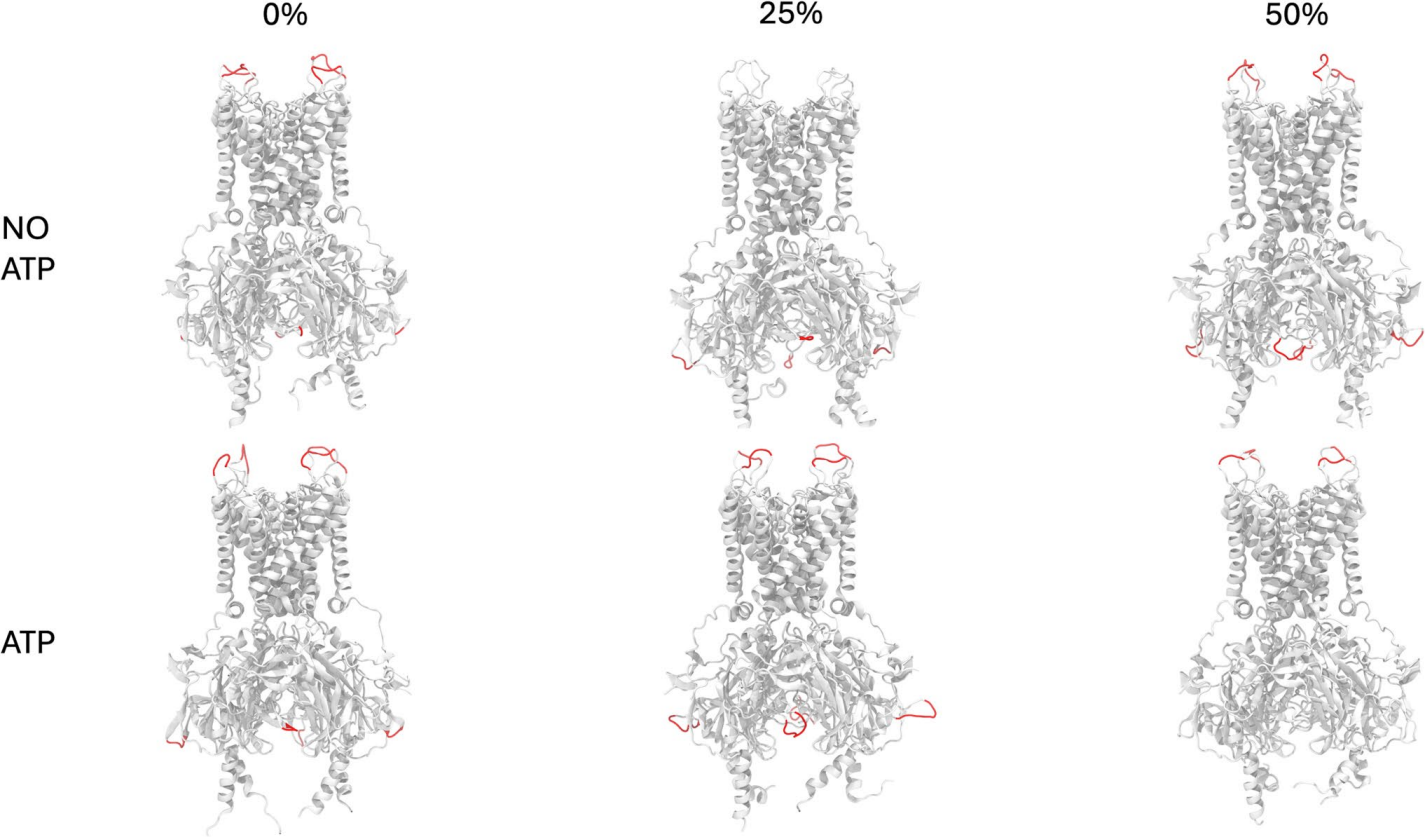
Root mean square fluctuations (RMSF) with value greater than 0.3 nm from Fig. 2 were mapped on their corresponding residues (Red). Membrane atoms were not shown

As noted on Fig. 2, Kir6.2 RMSF did not reflect the intermembrane behavior due to the membrane rigidity. As noted on Fig. 3, those residues with a RMSF value greater than 0.3 nm are located on loops (as expected) not closer to the membrane.

**Fig. 4:**
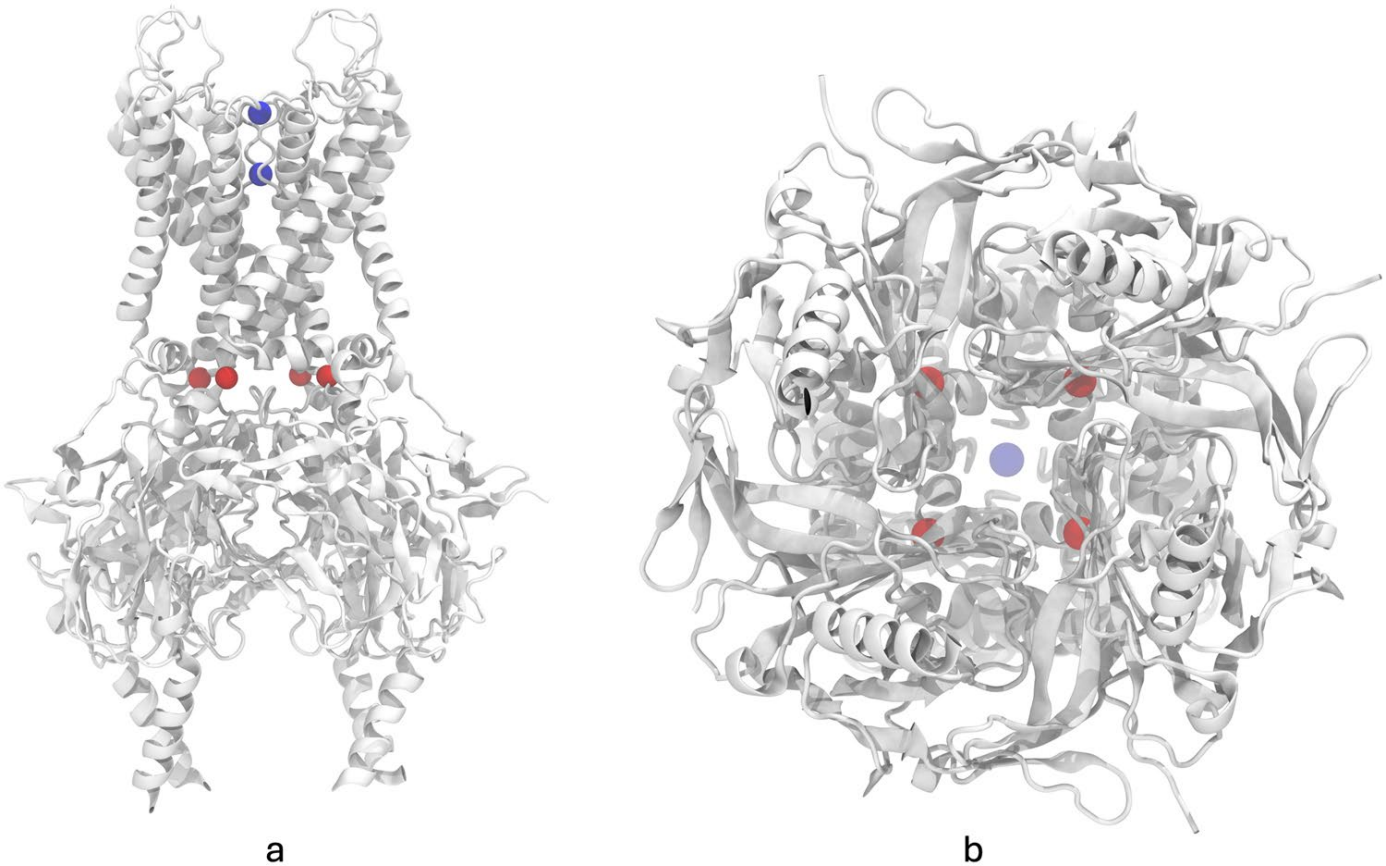
Two perspectives of Kir6.2 (a and b) with the original potassium ions (blue) for reference and four equivalent glutamine residues (red) (Q173)

To study the possible cholesterol effect on the channel, we analyzed aperture on its cytoplasmic side by monitoring the perimeter (~ 60 Å) formed by four equivalent glutamine residues (Q173) (Fig. 4). These α-carbons form an almost perfect square in the crystallographic structure, with distances between them measuring 15.51 ± 0.04 Å.

**Fig. 5:**
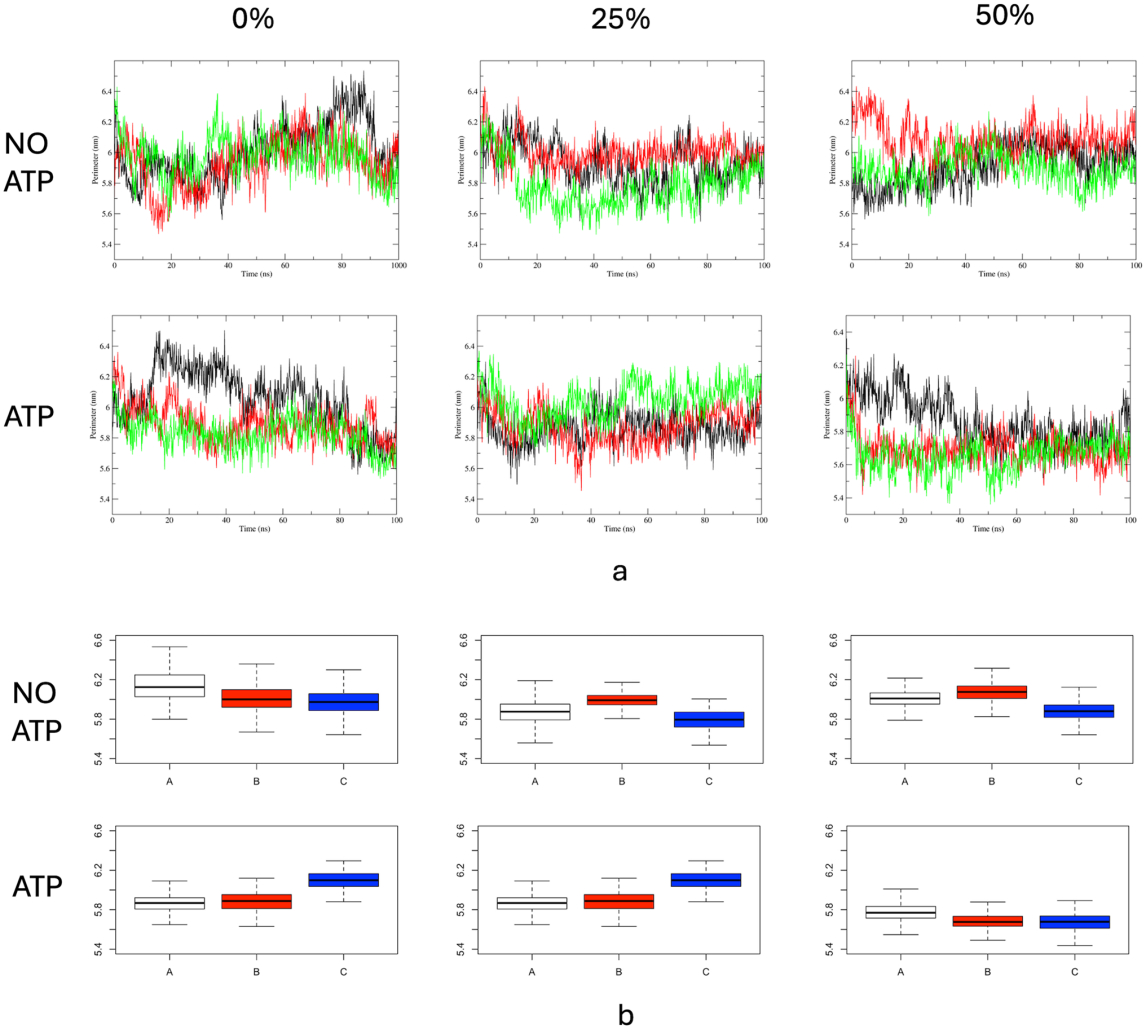
**a** Perimeter formed by four glutamine residues (Q173) over time for all the simulated time. **b** Quantitative summaries for the last 50 ns for each system (molecular dynamics equilibrated time). The average median for each system were NO ATP: 6.03 (0%), 5.90 (25%), 6.00 (50%) and ATP: 5.88 (0%), 6.00 (25%), and 5.71 (50%)

In addition, the perimeter formed by four equivalent glutamines (Q173 on each chain) exhibited larger fluctuations in replicates with 0% cholesterol, whereas the opposite was observed in replicates with 50% cholesterol (Fig. 5a). This effect was observed when the average median for the last 50 ns was analyzed (Fig. 5b). As noted, the average median for the 50% cholesterol had the lowest value (lowest perimeter value) of all the systems simulated. These results may indicate the effect of the cholesterol on the protein movement. The effect of cholesterol on membrane stiffness is a well-documented phenomenon (Doole et al. 2022; Raffy and Teissié 1999) (Fig. 5). Membrane stiffness appears to affect Kir6.2 by decreasing the aperture

### Binding Free Energy Calculations

Table 1 displays the electrostatic (ΔG_elec_) and non-electrostatic (ΔG_non-elec_) contributions to the binding free energy (ΔG_b_) for complexes derived from the cryo-EM structure of the Kir6.2 (7TYS) with ATP (Sung et al. 2022).

We determined the mean of the clusters over each condition to explain all the contributions to the binding energy between ATP and Kir6.2 in Table 1. As a hypothesis, we propose that increasing cholesterol content can lead to a more rigid membrane environment, which in turn can induce conformational changes in membrane proteins such as Kir6.2 and alter intermolecular interactions. ΔG_b_ can increase, making certain processes energetically less favorable at 25% cholesterol and then a decrease in ΔG_b_ at 50%.

On the other hand, Doole et al. 2022 reported that reduction of the area per lipid at the aqueous interface leads to greater bilayer thickness, in which an increased energetic penalty for separating or bending the lipids (increased area elastic modulus K_A_ and bending rigidity).

At 0% cholesterol, a ΔG_b_ = 53 kJ/mol was obtained; however, at 25% the ΔG_b_ increased (116 kJ/mol) and at 50% it decreased again (81 kJ/mol). This behavior agrees with and complements described by Hofsäss et al. (2003); they determined the volume and an area elastic (K_v_ and K_A_), respectively, moduli of the system from the fluctuations in volume and area of the entire system during the simulation on membrane at 0, 5, 10, 15, 25, and 40% cholesterol. It is important to mention that these parameters K_v_ and K_A_ were obtained by statistical mechanics which are directly related to the thermodynamic parameters K/ΔG_b_. Hence, Hofsäß C et al. 2003 obtained a zig-zag trend in K_A_ values, coinciding with an increasing value at 25% cholesterol and decreasing at 50% as our trend in ΔG_b_ values (Table 1).

The positive ΔG_b_ values in the study arise primarily from the solvation energy term, which indicates that desolvation (the process of removing water molecules from the interacting molecules) requires energy (Millán-Pacheco et al. 2023). This process involves rearranging water molecules around the binding partners (ATP and Kir6.2), which is often energetically unfavorable because the water molecules are tightly associated with the charged or polar groups of the molecules. In summary, while the positive ΔG_b_ values indicate that desolvation is energetically costly, the favorable Coulombic interactions can still make the binding interaction functionally important in a cellular context. The positive ΔG_b_ values simply highlight that the interaction might not be entirely spontaneous, but still biologically significant and relevant for cellular processes.

Electrostatic interactions are stronger at 0% cholesterol, leading to more favorable binding, but become weaker with increasing cholesterol at 25% and 50%. Besides, the distance between charged particles (ATP and Kir6.2) affects Coulombic interactions, and the conformational changes in internal loops of Kir6.2 in the presence of cholesterol contribute to this (Fig. 3). Furthermore, the trend in ΔG_non-elec_ values could be a result of both altered conformations of Kir6.2 and changes in the structure of the lipid bilayer, which may affect hydrophobic and van der Waals interactions.

This analysis highlights the complex relationship between membrane composition (cholesterol), protein conformational changes, and the electrostatic and non-electrostatic interactions that dictate the binding energetics between ATP and Kir6.2.

### Increased Affinity of K_ATP_ Channels for ATP Could Produce Hyperinsulinemia

**Fig. 6:**
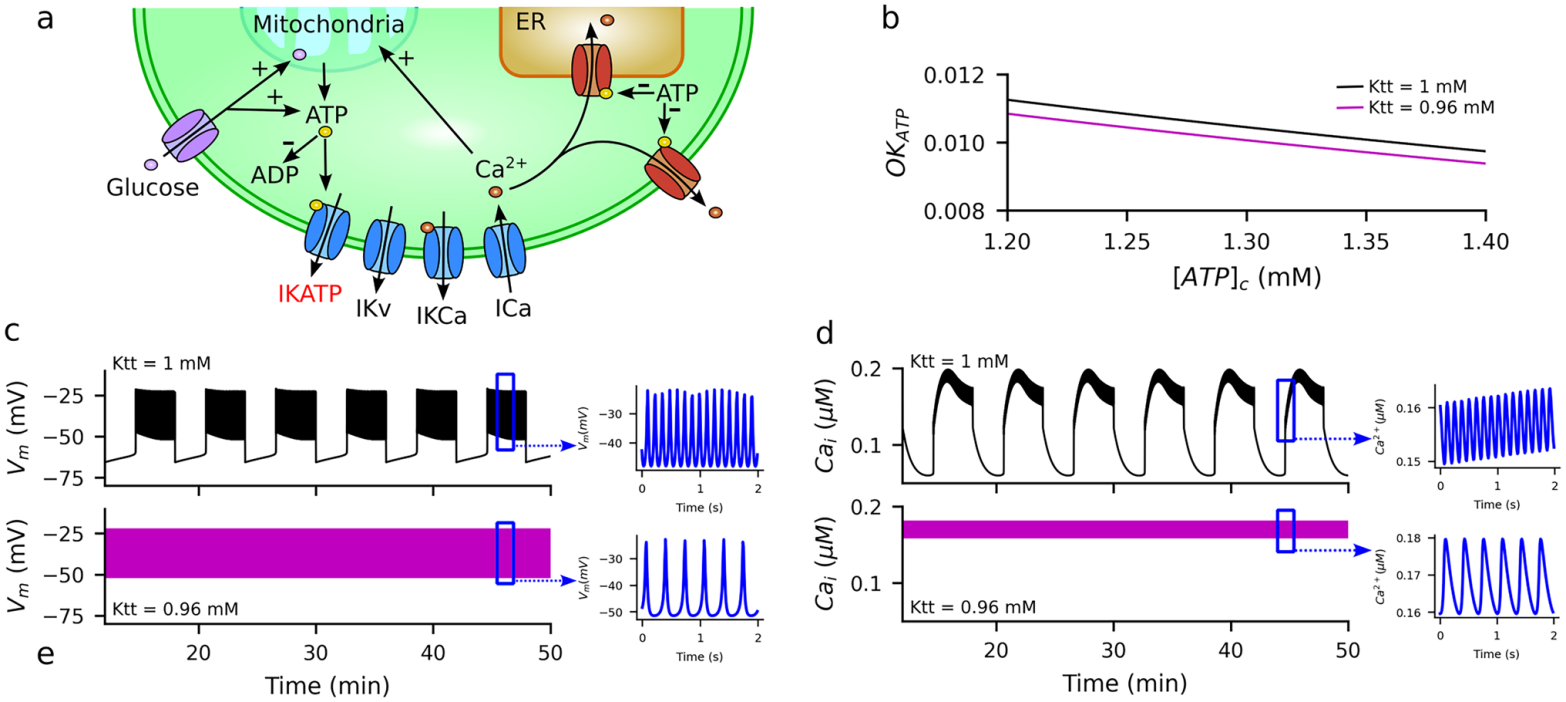
**a** Diagram of the Integrated Oscillator Model of the pancreatic beta-cell. Glucose transported into the cell is metabolized, driving the production of ATP. In response to the increase in intracellular ATP, K_ATP_ close leading to depolarization and the production of the electrical activity that leads to the increase in intracellular Ca^2+^, which further stimulates metabolism. ATP is consumed by the ATPases responsible for the uptake of Ca^2+^ into the endoplasmic reticulum (ER) and the extrusion of Ca^2+^ from the cell. Plus and minus signs indicate the mechanisms involved in the production and consumption of ATP, respectively. **b** Open probability of the K_ATP_ (OK_ATP_) as a function of intracellular ATP ([ATP]_c_). **c** Electrical activity of the pancreatic beta-cell for the normal and increased affinity to ATP of K_ATP_ channels. **d** Oscillations in intracellular Ca^2+^ [Ca]_i_. In b-d, the 4% increase in the affinity to ATP of K_ATP_ channels predicted by the molecular simulations was implemented in the IOM model by modifying the value of the dissociation constant Ktt from 1 to 0.96 mM

As described above in Table 1, the changes in ΔG_b_ produced by the increase in cholesterol concentration indicated a 4% increase in the affinity to ATP of the K_ATP_ channels. This was implemented in the IOM model of the beta-cell (Fig. 6a) by reducing the value of the corresponding affinity constant from Ktt = 1 mM to 0.96 mM, which reduced the open probability of the K_ATP_ channels (OK_ATP_) in the physiological range of ATP concentration (Fig. 6b). Consequently, the slight increase in ATP affinity significantly altered the functional behavior of beta-cells compared to conditions of normal affinity. This is illustrated in Figs. 6c and d, where the electrical activity and intracellular calcium (Ca_i_) are depicted for both the normal and increased affinity states, respectively. Normal affinity produced a typical slow electrical bursting within a period of several minutes, which underlies the calcium oscillations that ultimately drive the pulsatile nature of insulin secretion observed in healthy subjects (Satin et al. 2015). Slow bursting was accompanied by oscillations in intracellular calcium produced by the interplay between the production and consumption of calcium. Within the slow oscillatory behavior, high-frequency oscillations were produced in the membrane potential and intracellular Ca^2+^ (highlighted by the blue insets in Fig. 6c and d). In contrast, increasing the affinity of K_ATP_ for ATP in response to alterations in the cholesterol composition of the cell membrane, as suggested by the molecular simulations described before, led to a continuous increase in the electrical activity of the cell (Fig. 6c). Compared with the normal response, continuous spiking was accompanied by the abolition of calcium oscillations (Fig. 6d), showing high-frequency oscillations at stimulatory levels. Functionally, continuous spiking, accompanied by elevated intracellular calcium levels, leads to the continuous secretion of insulin, instead of the pulsatile secretion observed under normal conditions. Notably, the pulsatile nature of insulin secretion observed in healthy subjects is disrupted in patients with type 2 diabetes (Raffy and Teissié 1999), which, according to our simulations, could be attributed by the increased affinity to ATP. These results indicate that the increased affinity of K_ATP_ for ATP, likely due to alterations in the cholesterol composition of the beta-cell membrane, can lead to profound changes in the electrical activity of the cell. According to our simulations, these changes are expected to affect the dynamics of insulin secretion and likely to contribute to hyperinsulinemia observed in metabolic syndrome and early stages of type 2 diabetes.

Here, we have focused on the effects of cholesterol on the binding affinity of ATP to the K_ATP_ channel and the functional implications in the electrical activity of the pancreatic beta-cell. However, it has been reported that the function of other ion channels expressed in the membrane of the beta-cell, including inwardly rectifying ion channels, K^+^, Na^+^ and Ca^2+^ channels (Rorsman and Ashcroft 2018) could be also affected by changes in the cholesterol concentration (Levitan et al. 2010, 2014; Romanenko et al. 2004, 2002, 2014; Jiang and Levitan 2022). Although it has been reported that the most common effect of cholesterol on the activity of ion channels is to decrease the conductance, open probability and/or number of active channels on the cell membrane, the detailed mechanism underlying such effect has not been studied in detail (Levitan et al. 2010, 2014; Jiang and Levitan 2022). Future work should involve molecular simulations of other ion channels involved in the electrical activity of the pancreatic beta-cells to determine the extent of the effect of cholesterol on their electrophysiological properties, followed by functional simulations using mathematical models, such as the IOM model. On the other hand, validation experiments in which the cholesterol concentration in the plasma membrane of pancreatic beta-cells is tightly controlled remain technically challenging but are undoubtedly a matter of future work.

In summary, our simulations support the proposal that changes in membrane cholesterol in pancreatic beta-cells could have a profound impact on the regulation of insulin secretion by increasing the affinity to ATP of K_ATP_ channels. Overall, these insights provide a framework for understanding how membrane cholesterol might contribute to hyperinsulinism, a characteristic feature of metabolic syndrome or the early stages of type 2 diabetes.

## Data Availability

No datasets were generated or analyzed during the current study.
